# Can Herpesvirus Infections Influence When and How We Age?

**DOI:** 10.3390/v18101062

**Published:** 2026-09-24

**Authors:** Shilpa Deshpande Kaistha, Barry T. Rouse, L. C. Rodrigues Junior

**Affiliations:** 1 Department of Biotechnology, School of Life Sciences & Biotechnology, Chhatrapati Shahu Ji Maharaj University, Kanpur 208024, UP, India; shilpakaistha@csjmu.ac.in; 2 Department of Pathobiology, College of Veterinary Medicine, University of Tennessee, 1414 Cumberland Avenue, Knoxville, TN 37996, USA; btr@utk.edu; 3 Laboratory of Immunology, Department of Basic Health Science, Universidade Federal de Ciências da Saúde de Porto Alegre (UFCSPA), Porto Alegre 90050-170, Brazil

**Keywords:** herpesvirus, inflammaging, chronic inflammation, latency, immunosenescence, vaccine

## Abstract

Aging is accompanied by numerous interlinked cellular, genetic, and molecular changes, one of the most consequential being inflammaging: a chronic, low-grade systemic inflammatory state linked to metabolic dysregulation and pleiotropic effects on immune function. Although diverse intrinsic and extrinsic factors are known to drive inflammaging, the contribution of persistent viral infection remains incompletely understood. Here, we evaluate evidence that human herpesviruses, including cytomegalovirus (CMV), Epstein–Barr virus (EBV), herpes simplex viruses (HSV-1/2), and varicella zoster virus (VZV), may act as chronic antigenic and inflammatory stimuli capable of amplifying inflammaging and shaping the trajectory of aging. We review the molecular, cellular, and systems-level mechanisms by which herpesvirus latency and periodic reactivation sustain innate and adaptive immune activation, promote cellular senescence, and contribute to immune dysregulation and exhaustion. We further examine epidemiological evidence linking herpesvirus infection to dementia and other age-associated outcomes and highlight emerging data suggesting that antiviral treatment and vaccination, such as against VZV, may mitigate these effects. We conclude that herpesviruses are best understood as adjuvant contributors, rather than primary drivers, of inflammaging, and propose that persistent herpesvirus infection be considered an addressable risk factor in strategies aimed at promoting healthy aging.

## 1. Introduction

Aging is an inevitable event in mankind, and many throughout history have attempted to find ways of stopping or delaying its occurrence [1,2]. Much is now known as to why and how aging occurs and the topic remains a focus of research, with numerous excellent reviews describing our progress [3,4]. From discoveries in aging research to therapeutics for healthy aging, it is evident that a myriad of interacting events occurs during aging. These include genetic, molecular, cellular, physical, emotional and metabolic changes with the process affecting all tissues, organs and bodily functions, although some have more impact than others [5].

One prominent process associated with biological aging is inflammaging, a chronic, low-grade, systemic inflammatory state arising from reciprocal interactions among immunosenescence, cellular senescence, metabolic dysregulation and persistent immune activation [6]. The process is bidirectionally associated with aging, establishing a self-perpetuating cycle that promotes progressive tissue dysfunction and the development of age-related diseases [7,8]. Multiple inflammatory events occur during inflammaging, and characteristically, cells expressing a so-called pro-inflammatory senescence-associated secretory phenotype (SASP) become present, and these cells are considered involved in driving the process [9]. Many intrinsic and extrinsic events can lead to inflammaging, and, in this article, we make the case that persistent infections caused by human herpesviruses may act as cofactors or amplifiers of inflammaging that in turn accelerate the aging process.

Humans are infected with multiple types of herpesviruses, all of which can cause an acute, oftentimes silent infection that is followed by the virus persisting in the host for a prolonged and usually indefinite time in a status referred to as latency [10]. Depending on the virus, latency is associated with variable levels of viral gene expression, and in most instances, it can be reversed periodically, leading to reactivation, resumption of the productive infection cycle, and the generation of new viral progeny [11,12]. Human herpesviruses establish lifelong infections characterized by latency, intermittent reactivation, and long-term interactions with the host immune system. Although these viruses differ in their cellular and anatomical reservoirs, human cytomegalovirus (CMV), Epstein–Barr virus (EBV), herpes simplex virus type 1 (HSV-1), herpes simplex virus type 2 (HSV-2), and varicella zoster virus (VZV) can persist within the host and induce sustained or recurrent immune stimulation, potentially contributing to chronic inflammation and age-related immune dysfunction. Together, these features raise the possibility that lifelong herpesvirus persistence may contribute to inflammaging by repeatedly engaging immune pathways over the course of aging. In this article, we describe several mechanisms that could link human herpesvirus infections to inflammaging and consider whether controlling herpesvirus infection could potentially modulate this process. We examine the possibility that persistent herpesvirus infections may act as cofactors or amplifiers of inflammaging, thereby contributing to age-associated immune and tissue dysfunction.

## 2. Review Methodology

This narrative review was based on a focused literature search of PubMed/MEDLINE, Scopus, Web of Science, ScienceDirect, and Google Scholar, covering publications from 1993 to 2026. Relevant original studies, reviews, systematic reviews, meta-analyses, and epidemiological and mechanistic studies addressing human herpesvirus persistence, latency, reactivation, immune remodeling, immunosenescence, inflammation, inflammaging, and age-associated diseases were considered. Reference lists of relevant publications were also examined to identify additional studies.

Rather than providing a virus-by-virus systematic assessment, this review adopts an integrative approach to examine how CMV, HSV-1, HSV-2, EBV, and VZV may interact with common biological processes associated with inflammaging. Evidence was therefore synthesized comparatively, with emphasis on persistent viral activity, immune dysregulation, cellular stress and senescence, inflammatory signaling, and age-associated outcomes. The strength and limitations of the available evidence were considered when interpreting potential associations and mechanisms.

## 3. What Is Inflammaging and How Does It Relate to Aging?

Inflammaging refers to a chronic, low-grade inflammatory state that develops with aging and is characterized by persistent immune activation, dysregulated metabolic homeostasis, and a progressive decline in immune function [6]. Rather than being merely a consequence of aging, inflammaging is now recognized as one of its biological hallmarks. With age, the progressive accumulation of cellular and molecular damage activates innate immune sensing pathways, generating a persistent inflammatory environment that is associated with and may contribute to the aging process [13]. Prolonged exposure to a pro-inflammatory milieu can promote immune remodeling and contribute to an age-associated decline in immune competence. In vivo, recent evidence demonstrates that low-grade inflammation accelerates CD8^+^ T-cell aging and promotes the accumulation of age-associated granzyme K^+^ CD8^+^ T-cells. Notably, the same study showed that aged adipose tissue acts as a functional niche that provides tissue-derived signals supporting the maintenance and differentiation of age-associated CD8^+^ T-cells [14]. Immunosenescence may, in turn, reinforce inflammaging through impaired immune surveillance, reduced clearance of senescent cells, defective resolution of inflammation, and diminished control of latent infections. Experimental studies in aged mice have demonstrated impaired efferocytosis and defective resolution of inflammation, providing a potential mechanism through which impaired immune clearance may sustain inflammatory signaling [15]. Age-related impairment in immune surveillance may also favor the persistence and accumulation of senescent cells, further reinforcing inflammatory signaling [16]. Together, these mechanisms may contribute to a self-reinforcing cycle linking inflammaging, immune dysfunction, and biological aging.

An important contributor to inflammaging is the accumulation of senescent cells exhibiting the SASP, a heterogeneous, pro-inflammatory and tissue-remodeling secretome comprising cytokines (e.g., IL-6, TNF-α, IL-1β), chemokines, matrix metalloproteinases (MMPs), growth factors, and other bioactive molecules [9]. The NF-κB pathway centrally regulates inflammatory gene expression and is critical for maintaining the SASP [17,18]. Acting in an autocrine and paracrine manner, the SASP reinforces senescence and promotes extracellular matrix degradation and metabolic dysfunction in neighboring tissue [19]. Mitochondrial dysfunction is also closely intertwined with this process. Senescent and metabolically stressed cells can exhibit increased mitochondrial reactive oxygen species (ROS) production and accumulation of damaged mitochondria, which may promote NLRP3 inflammasome activation [20,21]. Additionally, mitochondrial damage can result in the release of mitochondrial DAMPs (mtDAMPs), including oxidized mitochondrial DNA (mtDNA), into the cytosol, where they can activate NLRP33 [20,22]. Progressive exposure to endogenous danger signals and other inflammatory stimuli over the course of life may progressively remodel immune function, contributing to immunosenescence and a persistent state of low-grade inflammatory activation [3,23,24].

While inflammaging is largely associated with endogenous damage signals and age-related tissue and immune dysfunction rather than overt infection, persistent antigenic exposure to microbial and environmental stimuli may also contribute to age-related immune remodeling and dysfunction [6]. Together with episodic reactivation of latent viruses, such antigenic stimulation may help sustain chronic, low-grade inflammation even in the absence of clinically apparent infection [6,25,26]. Within this framework, persistent virus–host interactions have emerged as potential extrinsic modifiers of inflammaging. Herpesviruses are particularly relevant among persistent viral infections because they establish lifelong latency punctuated by episodes of reactivation. Importantly, low-level viral gene expression or localized “micro-reactivation” events may occur without overt viral disease, potentially providing intermittent immune stimulation over the lifespan. Beyond overt reactivation, persistent herpesvirus infection requires continuous immune surveillance, potentially imposing a sustained immunological burden. This is particularly evident for CMV, in which long-term antigenic stimulation is associated with marked expansion of virus-specific effector T-cell populations, a phenomenon termed “memory inflation” [27]. Experimental studies have provided substantial evidence that persistent CMV infection reshapes the CD8^+^ T-cell compartment, driving the accumulation of differentiated effector memory cells, contraction of the naïve CD8^+^ T-cell pool, and impaired responses to heterologous infections, features closely associated with immunosenescence [28,29]. A comparable, though less pronounced, pattern of antigen-driven T-cell remodeling has been described for EBV, in which persistent infection sustains expanded populations of EBV-specific CD8^+^ T-cells that progressively acquire senescence-associated phenotypic and functional features over the lifespan [30]. Persistent HSV-1 infection, in turn, has been shown to promote localized neuroinflammation and senescence-related changes in the aging nervous system, while experimentally induced HSV-1 reactivation triggers innate immune activation and inflammatory cell recruitment at the site of recurrent infection [31,32]. For VZV, experimental and clinical studies indicate that reactivation is associated with localized inflammatory responses and impaired virus-specific cellular immunity, particularly with aging, although its broader contribution to age-related immune remodeling remains less well established [33,34]. This raises the question of how latency itself, as a biological state, may shape the consequences of reactivation and their potential cumulative impact on the aging immune system.

## 4. Human Herpesviruses: General Aspects of Latency and Reactivation and Their Impact on Inflammaging

The lifelong persistence of human herpesviruses has garnered increasing attention as a potential contributor to age-associated immune remodeling and chronic low-grade inflammation [35,36]. Unlike classical chronic viral infections such as human immunodeficiency virus (HIV) and hepatitis B virus (HBV), in which viral persistence is generally associated with ongoing or sustained viral replication and prolonged antigen exposure, members of the *Herpesviridae* family establish lifelong persistence primarily through latency, while retaining the capacity for periodic reactivation throughout the host’s lifetime [10].

During primary infection, productive viral replication triggers broad innate immune activation. Viral components are detected by pattern recognition receptors including Toll-like receptors (TLRs), cyclic GMP-AMP synthase–stimulator of interferon genes, RIG-I-like receptors, and inflammasomes. Activation of these pathways induces type I and III interferons, pro-inflammatory cytokines, and chemokines that orchestrate the early antiviral response [37,38,39]. Following the establishment of latency, however, the viral genome persists within host cells with highly restricted viral gene expression as an episome [40,41,42]. The classical view of herpesvirus latency as a quiescent, biologically silent state has been progressively replaced by a model in which latency is a dynamic equilibrium between viral activity and host immune control [43,44,45]. Maintenance of latency requires coordinated viral and host mechanisms, including epigenetic regulation of viral gene expression [46], metabolic adaptation of infected cells [47], mitochondrial pathway changes [48], and continuous immune surveillance. Virus-specific cytotoxic T lymphocytes, natural killer (NK) cells, antiviral cytokines, and cell-intrinsic mechanisms collectively constrain viral gene expression and prevent productive replication. Thus, although viral replication may be undetectable during latency, the host continues to invest substantial immunological and cellular resources in controlling the persistent infection.

This viral–host equilibrium may become progressively destabilized with aging as multiple age-associated alterations compromise antiviral immune surveillance and cellular homeostasis [49]. Immunosenescence, mitochondrial dysfunction, increased oxidative stress, epigenetic drift, impaired autophagy, and the accumulation of senescent cells can collectively reshape the cellular and tissue environment, potentially impairing viral control and creating conditions that favor viral persistence, gene expression, or reactivation [50,51]. The molecular triggers and cellular checkpoints governing reactivation differ substantially among herpesvirus species. In HSV-1 and HSV-2, reactivation from sensory neurons can be triggered by physical or psychological stress, ultraviolet exposure, fever, and local immunosuppression, which may disrupt epigenetic repression of the viral genome [52,53]. VZV reactivation, clinically manifesting as herpes zoster, is strongly associated with age-related reductions in VZV-specific CD4^+^ and CD8^+^ T-cell immunity, allowing for the virus to escape long-term control within sensory ganglia [54]. EBV reactivation in memory B-cells is closely linked to plasma cell differentiation and accompanied by changes in the expression of latency-associated genes. This process may be promoted by immunosuppression or co-infection, which can disrupt immune control of latent EBV infection [55]. CMV, in contrast, can reactivate during myeloid cell differentiation toward macrophages, a process influenced by inflammatory cytokines and immunosuppressive conditions [56]. These distinct persistence and reactivation strategies highlight the diverse ways in which herpesviruses may contribute to age-associated immune dysfunction.

Importantly, persistent herpesvirus infection may involve intermittent low-level viral gene expression or subclinical reactivation events that provide episodic antigenic stimulation and sustain virus-specific immune responses over prolonged periods [27,57]. Repeated episodes of viral antigen exposure may therefore impose a cumulative immunological burden over decades. CMV provides the strongest example of this phenomenon, with recurrent antigenic stimulation associated with memory inflation, expansion of differentiated T-cell populations, and features of immune exhaustion or dysfunction [58]. Comparable effects are less firmly established for other human herpesviruses and remain an important area of investigation.

Intermittent low-level viral activity during latency or subclinical reactivation may provide repeated antigenic stimulation, engaging antiviral immune surveillance and contributing to sustained immune activation [59]. With aging, the progressive decline in antiviral immune control may increase the frequency or persistence of such viral activity, allowing for recurrent immune stimulation to interact with age-associated inflammatory and cellular stress pathways. Rather than acting as independent drivers of inflammaging, herpesviruses may therefore function as context-dependent cofactors that reinforce pre-existing age-related inflammatory processes. The magnitude and nature of this contribution are likely to vary according to viral species, tissue reservoir, reactivation dynamics, and host immune status. Although the relative contributions of latent viral activity, low-level viral gene expression, and overt reactivation remain incompletely understood, these mechanisms may collectively contribute to a persistent inflammatory burden over the course of aging. Figure 1 summarizes a proposed conceptual model in which lifelong herpesvirus persistence, age-related weakening of antiviral immune control, and intermittent viral activity interact to promote sustained immune activation and potentially reinforce inflammaging.

## 5. Herpesvirus-Specific Contributions to Inflammaging: A Comparative Appraisal

In this section, we examine the potential mechanisms by which CMV, HSV-1, HSV-2, EBV, and VZV may contribute to inflammaging. Other human herpesviruses, including HHV-6A/6B, HHV-7, and HHV-8, also establish persistent infections and may influence immune and inflammatory processes; however, their specific contributions to aging and inflammaging remain less extensively characterized. Rather than reiterating common features of herpesvirus biology, we emphasize the characteristics that may distinguish the contribution of the five major human herpesviruses, including the anatomical and immunological compartments involved, the mechanisms governing persistence and reactivation, and the strength and quality of the available evidence. Importantly, most studies to date have investigated individual herpesviruses separately, often in different populations, clinical settings, or experimental conditions, limiting direct comparisons of their relative contributions to immune aging. This represents an important gap, particularly because infection with multiple herpesviruses is common over the human lifespan, meaning that their effects on immune function and inflammation may occur concurrently within the same host. Accordingly, a comparative perspective may provide insights that are not apparent when each virus is considered in isolation. We therefore assess not only the evidence linking individual herpesviruses to inflammaging and age-related dysfunction, but also the extent to which their distinct persistence and reactivation strategies may differentially, or potentially cumulatively, shape the aging immune environment. Finally, we discuss the extent to which the available evidence supports a causal, rather than merely associative, relationship between herpesvirus activity, inflammaging, and age-related dysfunction.

### 5.1. Cytomegalovirus Virus (CMV)

Among the human herpesviruses, CMV has the strongest body of evidence linking persistent infection to age-associated immune remodeling [60,61]. A major feature underlying this association is the sustained antigenic pressure imposed by lifelong CMV infection, reflected by the progressive expansion of CMV-specific T-cell populations over decades of infection, the phenomenon of memory inflation [62]. This process can reshape the T-cell compartment through the accumulation of highly expanded and oligoclonal CMV-specific clones, raising the possibility that persistent CMV-driven expansion may alter the availability and functional composition of the T-cell repertoire for responses to unrelated antigens during aging [63]. At the cellular level, memory inflation is accompanied by the accumulation of highly differentiated CD8^+^ T-cells characterized by phenotypic features associated with replicative history and immune aging, including loss of CD28 and increased expression of CD57 and KLRG1 (CD28^−^, CD57^+^, KLRG1^+^) [64,65]. These changes may reduce proliferative capacity and functional plasticity while favoring the persistence of terminally differentiated effector populations.

Beyond T-cell remodeling, chronic or recurrent CMV reactivation can engage monocytes, NK cells, endothelial cells, and tissue-resident immune populations, promoting the production of TNF-α, IL-6, IFN-γ, and other pro-inflammatory mediators. Persistent activation of these cellular and inflammatory pathways may contribute to low-grade systemic inflammation and reinforce age-associated immune dysfunction [66,67]. Consistent with this association, data from the MARK-AGE cohort of 2065 individuals showed that higher CMV IgG titers were associated with elevated inflammatory markers, including homocysteine, ceruloplasmin, and α-2-macroglobulin, as well as increased oxidative stress and reduced levels of antioxidant factors such as α-tocopherol, selenium, and vitamin D [68].

CMV infection also induces extensive metabolic remodeling in infected cells, involving mitochondrial function, oxidative metabolism, and glucose utilization, with consequences for both viral replication and cellular inflammatory responses [69,70]. Rather than simply increasing general cellular metabolism, CMV establishes a virus-specific metabolic program encompassing glycolysis, glutaminolysis, the tricarboxylic acid (TCA) cycle, nucleotide biosynthesis, and lipid synthesis [71]. Importantly, these metabolic adaptations are dynamic and temporally regulated, with early mitochondrial biogenesis followed by broader anabolic remodeling that supports the energetic, nucleotide, and membrane requirements of viral replication [72,73]. This metabolic reprogramming may also increase cellular stress, particularly through alterations in mitochondrial electron transport and redox homeostasis [74]. Increased mitochondrial respiratory activity can enhance electron leakage and superoxide generation, which is subsequently converted to H_2_O_2_ by mitochondrial superoxide dismutase (SOD2) [75]. H_2_O_2_ can, in turn, promote CMV lytic replication in fibroblasts through extracellular and intracellular mechanisms, whereas antioxidant pretreatment inhibits viral replication. Mechanistically, activation of the p38-MAPK pathway contributes to H_2_O_2_-mediated stimulation of HCMV replication [76]. These findings suggest a potential feed-forward interaction in which CMV-driven metabolic and mitochondrial remodeling increases redox signaling, which may subsequently promote viral replication and sustain cellular stress and inflammatory pathways.

In the context of chronic CMV stimulation, sustained inflammatory and metabolic stress maybe associated with cellular dysfunction and, under specific conditions, promote the acquisition of senescence-associated features [68]. Senescent cells can subsequently acquire SASP, characterized by the production of inflammatory cytokines, chemokines, and other mediators capable of modifying the surrounding tissue environment [9]. This provides a potential mechanistic link between CMV persistence, cellular stress, and chronic inflammation. However, whether CMV directly drives or accelerates cellular senescence and SASP in vivo remains insufficiently established. These mechanisms should therefore be considered as potential contributors that may overlap with, and reinforce, pathways of immunosenescence and inflammaging driven by intrinsic aging processes that require stronger direct evidence [26,65].

Although the causal directionality of these interactions remains difficult to establish, the convergence of immune, metabolic, and cellular evidence places CMV in a distinct position among human herpesviruses, with a broad and mechanistically plausible contribution to age-associated immune dysfunction. Figure 2 summarizes the interconnected mechanisms through which persistent CMV infection may contribute to immune remodeling, cellular stress, and inflammaging.

### 5.2. Herpes Simplex Virus (HSV-1 and HSV-2)

In contrast to CMV, whose influence on immune aging is largely systemic, HSV-1 and HSV-2 exert their effects through a more anatomically constrained, but biologically complex network involving neuronal, immune, and potentially lymphoid reservoirs. Following primary infection, both HSV-1 and HSV-2 establish latency primarily in sensory ganglia, where cycles of quiescence and reactivation continue throughout life. Each reactivation event, whether symptomatic or subclinical, exposes host tissues to intermittent viral gene expression and antigenic stimulation, potentially contributing to episodic inflammatory responses [77,78]. Whether these events contribute to a cumulative inflammatory burden that gradually builds over decades remains uncertain and largely speculative, as direct longitudinal evidence supporting this model in humans is limited. Importantly, clinically, HSV-1 and HSV-2 recurrences generally decline in frequency over time after primary infection, although substantial inter-individual variability exists and subclinical viral shedding may persist [79].

Emerging evidence suggests that HSV persistence may extend beyond sensory neurons. Recent experimental studies have detected HSV-1 genomes in non-neural, immune-rich lymphoid tissues and provided evidence of stress-induced viral reactivation from these sites in animal models, raising the possibility that such tissues may represent additional reservoirs of persistent infection [80,81]. However, whether such non-neuronal reservoirs represent bona fide latency or another form of viral persistence, and what their contribution to viral maintenance and disease may be, remains unresolved. If confirmed, a broader reservoir landscape would imply that HSV persistence engages immune surveillance mechanisms across multiple anatomical sites, with implications for the magnitude and chronicity of associated inflammatory signaling.

Productive HSV reactivation can be associated with injury or death of infected cells, generating episodic tissue injury and inflammatory responses involving infected and neighboring cells. Such responses provide a potential, but not yet established, mechanistic bridge between viral persistence and chronic inflammation. In neurons, viral manipulation of mitochondrial homeostasis may also contribute to the transition from latency to productive infection, as exemplified by the effects of the HSV-1 UL12.5 protein, which has been reported to contribute to mitochondrial nucleic acid stress and efficient viral reactivation [82]. Beyond structural alterations, HSV-1 UL12 encodes a conserved alkaline nuclease that gives rise to the amino-terminally truncated mitochondrial isoform UL12.5. UL12.5 localizes to mitochondria and triggers rapid and extensive depletion of host mitochondrial DNA during productive infection. Importantly, this process does not appear to depend exclusively on the intrinsic nuclease activity of UL12.5, with host mitochondrial nucleases such as ENDOG and EXOG also contributing to mtDNA degradation [83]. These mitochondrial perturbations provide a plausible mechanistic link to innate immune activation and cellular stress. However, whether they contribute to SASP-mediated inflammaging in vivo remains unresolved.

Taken together, the available evidence supports a mechanistic model in which recurrent HSV reactivation, mitochondrial dysfunction, innate immune activation, cellular stress and senescence, and neuroimmune signaling may converge to promote episodic and potentially persistent inflammatory responses during aging. The principal mechanisms proposed that may link HSV persistence with inflammaging are summarized in Figure 3, while emphasizing that several components of this model remain hypothetical and require validation in longitudinal human studies.

The neuroimmune dimension of HSV-1 has attracted particular attention in the context of brain aging. Following primary infection, HSV-1 can access the central nervous system through both trigeminal and olfactory pathways and HSV-1 DNA has been detected in human nervous tissues [84]. In a detailed mouse model, the brainstem served as the primary CNS entry point via the trigeminal connection, with infection detected in serotonergic and adrenergic neuromodulatory centers and hypothalamic nuclei, while the olfactory bulb showed relatively little viral antigen [85]. Although productive CNS infection is rare under normal immunological conditions, experimental evidence indicates that HSV-1 persistence and recurrent reactivation can stimulate local innate immune responses, including microglial activation, type I interferon signaling, and production of pro-inflammatory mediators. Repeated activation of these pathways may contribute to a neuroinflammatory environment resembling processes implicated in age-related neuroinflammation [86,87]. In this context, HSV-1 is best understood not only as an occasional cause of neurodegeneration, but as one potential component of a multifactorial process in which recurring inflammatory stimuli may progressively lower neuronal resilience and disturb tissue homeostasis in a brain already subject to the cumulative pressures of aging. A seminal study linking HSV-1 to Alzheimer’s disease (AD) demonstrated the presence of latent HSV-1 DNA in human brain tissue and reported that APOE-ε4 carriers with HSV-1 DNA in the brain had a substantially increased risk of AD [88]. Experimental models have shown that recurrent HSV-1 infection and reactivation can induce several AD-associated pathological features, including amyloid-β accumulation and deposition, tau hyperphosphorylation, synaptic dysfunction, neuroinflammation, and oxidative damage [86,89].

Several epidemiological studies have reported associations between HSV-1-related immune markers and AD and other forms of dementia; however, the findings have been inconsistent across studies [90,91,92]. This AD association was subsequently supported in the prospective Bordeaux-3C cohort, in which HSV-1 serological markers consistent with more frequent reactivation were associated with increased AD risk specifically among APOE-ε4 carriers [93]. Recently, a VALAD randomized clinical trial found that 78 weeks of valacyclovir treatment did not improve cognitive or functional outcomes in patients with early symptomatic AD and HSV-1/2 seropositivity. Notably, cognitive decline on the primary outcome was greater in the valacyclovir group than in the placebo group, with no significant effects on amyloid or tau PET measures. These findings argue against the use of valacyclovir as a disease-modifying treatment for established AD, while not excluding a potential role for HSV in earlier stages of disease development or prevention [94]. The trial, however, was not designed to assess systemic inflammaging, age related inflammatory trajectories, or the consequences of lifelong suppression of HSV reactivation. Thus, the results argue against a simple causal model linking ongoing HSV replication to established AD, but do not by themselves exclude a context dependent contribution of HSV persistence or intermittent reactivation to inflammatory remodeling during aging.

HSV-2 has received considerably less attention than HSV-1 in relation to biological aging and neurodegeneration, but emerging epidemiological evidence suggests that persistent infection is related to some neurological disorders. In a population-based cohort of 1022 older adults, higher HSV-2 IgG levels were associated with poorer cognitive performance at baseline increasing over five years with decline in memory domains [95]. In addition, an imaging study of 455 dementia-free older adults reported HSV-2 serological titers with reduced whole-brain cortical thickness, although HSV-2 exposure was not associated with hippocampal volume or an AD specific cortical thickness signature [96]. However, in contrast, a 2026 systematic review and meta-analysis of seven independent studies comprising 751,156 individuals found no significant association between HSV-2 and AD and all-cause dementia when odds ratios were pooled [97]. Thus, HSV-2 may represent a potential modifier of cognitive and brain decline, but whether recurrent HSV-2 reactivation contributes to inflammaging, neuroinflammation, or dementia through a biological pathway remains unresolved.

Taken together, HSV-1 and HSV-2 can be viewed as components of a broader host–virus ecosystem that influences the immune aging. Their recurrent reactivation is associate with sustained immune surveillance requirements, while potential engagement of neuronal and non-neuronal compartments provide biologically plausible, although not yet conclusively established, mechanisms through which persistent viral activity may contribute to inflammatory remodeling during aging. As illustrated in Figure 3, these processes are unlikely to operate independently but rather form an interconnected network linking recurrent viral reactivation with mitochondrial dysfunction, innate immune activation, neuroinflammation, and cellular senescence. Defining the relative contribution and mechanistic significance of these pathways remains an important challenge for future research.

### 5.3. Epstein–Barr Virus (EBV)

EBV occupies a biologically distinctive position among human herpesviruses by establishing lifelong latency within memory B-cells, the very compartment that sustains long-term humoral immunological memory [98]. This creates an unusually intimate host–virus relationship, in which viral persistence is embedded within the architecture of adaptive immunity rather than restricted to specialized anatomical reservoirs. Given the near-universal prevalence of EBV and its profound influence on B-cell biology, this raises an intriguing question: could a virus that persists within the memory compartment itself contribute to the remodeling of immune function over the course of aging? Despite its ubiquity and biological importance, however, EBV remains considerably less explored than CMV in the context of inflammaging and immunosenescence.

The intimate association of EBV with B-cell biology extends to cellular metabolism. EBV infection induces extensive metabolic reprogramming, including increased glycolysis, oxidative phosphorylation, one-carbon metabolism, and redox remodeling, supporting B-cell proliferation and viral transformation [99]. Recent evidence further demonstrates that EBV enhances cardiolipin biosynthesis through an EBNA2/MYC-dependent mechanism, promoting mitochondrial cristae biogenesis and sustaining mitochondrial respiration, one-carbon metabolism, and aspartate synthesis in infected B-cells [100]. These metabolic adaptations are primarily understood as mechanisms that support the energetic and biosynthetic demands of EBV-infected cells. Nevertheless, the possibility that persistent viral manipulation of mitochondrial and metabolic pathways may have broader consequences for immune cell homeostasis is particularly intriguing in the context of lifelong infection. Chronic alterations in metabolic and redox states could potentially intersect with cellular stress, immune differentiation, and inflammatory signaling, thereby providing a plausible, but still unproven, link between EBV persistence and age-associated immune dysfunction. Whether such metabolic remodeling has consequences beyond infected cells and whether it contributes meaningfully to systemic inflammaging remain important unresolved questions. The potential interplay between EBV persistence, metabolic and mitochondrial remodeling, immune cell differentiation, and age-associated inflammatory dysfunction is summarized in Figure 4.

Early models of immunosenescence proposed that persistent herpesvirus infections could contribute to the progressive remodeling of the T-cell compartment, including the accumulation of oligoclonally expanded, late-differentiated populations. EBV-specific T-cell responses were subsequently considered within this broader framework, although the evidence for herpesvirus-driven immunological space occupation has been substantially stronger and more consistent for CMV [101,102,103]. EBV-specific CD8^+^ T-cells nevertheless undergo marked remodeling across the lifespan. Rather than a uniform loss of function, aging appears to be associated with selective shifts in clonality, differentiation state, and functional capacity, with substantial EBV-specific effector responses often retained in otherwise healthy older adults [104,105,106]. Thus, EBV may not simply impose a progressive depletion of immune competence; instead, its lifelong presence may help shape the composition and functional architecture of antiviral memory.

This perspective becomes particularly interesting when considering the qualitative nature of EBV-specific T-cell remodeling. Recent evidence indicates that EBV-specific CD8^+^ T-cells can follow clonally imprinted differentiation trajectories accompanied by the acquisition of innate-like functional programs and persistent engagement of inflammatory signaling pathways [30]. Such findings raise the possibility that repeated or sustained immune surveillance of a lifelong latent virus may leave a durable imprint on T-cell differentiation without necessarily resulting in global immune exhaustion. In this sense, EBV may represent a model of immune adaptation to persistent antigenic experience, in which the consequences of lifelong viral persistence are expressed not primarily as immune failure, but as progressive remodeling of immune cell states and repertoires.

Rather than being regarded as a primary driver of inflammaging, EBV may therefore be better conceptualized as a potential modifier of age-related immune trajectories. Its effects are likely to depend on the interaction between viral persistence and host factors, including immune competence, genetic background, co-infections, and the broader inflammatory environment. This context dependence may be particularly relevant beyond the peripheral immune system. EBV has been investigated in relation to neuroinflammatory and neurodegenerative processes through its effects on B-cell biology and immune regulation, while viral latency programs can engage pathways such as NF-κB that influence inflammatory signaling [107]. Emerging evidence has also raised the possibility that EBV-associated immune perturbations may intersect with blood–brain barrier integrity and neuroinflammatory pathways, although direct causal links between EBV infection and dementia remain incompletely established [108]. The broader significance of EBV in immune aging may lie less in a direct virus–disease relationship than in a fundamental question: how does lifelong persistence within the memory immune compartment reshape immune aging? Determining whether EBV is a passenger, modifier, or active contributor to age-related inflammatory and neuroimmune changes remains an important challenge for future research.

### 5.4. Varicella Zoster Virus (VZV)

VZV offers a particularly instructive model for examining the bidirectional relationship between viral persistence and immune aging. This is not because its contribution to systemic inflammaging is well established, but because the consequences of declining immune control over latent VZV infection are among the most clinically evident manifestations of herpesvirus reactivation. Following primary infection, VZV establishes lifelong latency predominantly in sensory neurons of the dorsal root ganglia and may reactivate intermittently throughout life. Although many episodes of viral reactivation may remain clinically inapparent, clinically overt reactivation manifests as herpes zoster, whose incidence increases markedly with advancing age as VZV-specific cellular immunity declines [109].

A hallmark of immune aging is the progressive decline in VZV-specific cellular immunity, particularly within the CD4^+^ T-cell compartment, which becomes increasingly evident from middle age onward. This decline is considered an important determinant of the increased susceptibility to herpes zoster observed in older adults [110]. Reduced frequency and functional impairment of VZV-specific memory CD4^+^ T-cells may weaken immune surveillance of latent virus, thereby increasing the likelihood of viral reactivation from neuronal reservoirs and subsequent local and potentially systemic immune activation [111]. The importance of this cellular immune axis is further underscored by the ability of zoster vaccination to boost VZV-specific cellular immune responses in older adults [112,113]. In addition, a large quasi-experimental study found that herpes zoster vaccination was associated with a substantial reduction in the risk of dementia among older adults [114]. The proposed relationship between age-associated decline in VZV-specific immunity, intermittent viral reactivation, immune activation, and the resulting inflammatory and neurological consequences is summarized in Figure 5. These observations highlight the central role of age-associated changes in VZV-specific immunity in shaping the long-term host–virus relationship and suggest that declining immune surveillance may represent one mechanism through which persistent viral infections interact with biological aging.

Beyond its well-recognized neurological manifestations, VZV reactivation can engage inflammatory pathways in both neural and peripheral tissues. Clinical and experimental studies indicate that VZV reactivation is accompanied by transient inflammatory responses, including increased production of mediators such as IL-6, TNF-α, and IFN-γ, together with recruitment and activation of VZV-specific immune cells in affected tissues [115,116] . Experimental studies show that VZV activates innate immune signaling in human cells, including TLR2-dependent NF-κB activation and production of inflammatory mediators such as IL-6 and TNF-α [117], while clinical studies of herpes zoster demonstrate recruitment and activation of VZV-specific T-cells, NK cells, and monocytes at affected sites [118]. VZV reactivation can also involve cerebral vasculature. Thus, VZV vasculopathy is an established complication that may cause ischemic stroke and other cerebrovascular lesions, particularly in older or immunocompromised individuals [119]. More recently, prospective epidemiological evidence has linked a history of herpes zoster to an increased long-term risk of subjective cognitive decline, although whether this association reflects direct neuroinflammation, vascular injury, or shared susceptibility remains uncertain [120].

Taken together, the findings on VZV provide biological plausibility for a contribution of recurrent VZV reactivation to age-related inflammatory and neurological pathology, although direct evidence that VZV persistence drives sustained systemic inflammaging remains limited. As with the other herpesviruses, an important unresolved question is whether VZV reactivation actively contributes to age-related immune and tissue dysfunction, but instead primarily reflects the progressive loss of immune control that accompanies aging. Resolving this distinction will be essential to determine whether VZV represents a modifiable contributor to age-related inflammatory burden or mainly a marker of immunological vulnerability. Collectively, CMV, EBV, VZV, and HSV-1/2 occupy distinct cellular reservoirs and act through largely non-overlapping mechanisms.

Among the herpesviruses, the case for an active contribution to inflammaging is strongest for CMV; EBV, VZV, and HSV-1/2 remain plausible but less firmly established modulators, whose relative importance likely depends on host genetics, immune competence, and cumulative antigenic exposure over the lifespan. Although the evidence differs substantially among these viruses. The relative contribution to inflammaging remains uncertain and likely depends on host genetics, immune competence, and cumulative antigenic exposure over the lifespan.

## 6. Conclusions and Future Directions

In most developed countries, populations are aging, and there is growing interest in delaying the onset and impact of age-related decline. Achieving this goal requires understanding why and how aging occurs, since this knowledge may eventually allow for its course to be modified by addressing specific contributing factors. This review examined the possibility that herpesvirus infections can contribute to inflammaging, considering the available evidence, proposed mechanisms, and potential avenues for intervention. As the late herpes virologist Bernard Roizman observed, “Unlike love, herpes is forever”. Indeed, older individuals are chronically infected, usually sub-clinically, with multiple herpesviruses, making it inherently difficult to implicate any single infection as a direct cause of aging. Rather, herpesviruses likely act as cofactors or modifiers of age-related processes during persistent infection and episodes of viral activity, representing an additional source of immune and inflammatory pressure superimposed on the physiological changes of aging.

The strength of evidence differs substantially among the five herpesviruses discussed, with CMV the most consistently implicated in age-associated immune remodeling, while EBV, HSV-1, HSV-2, and VZV show more heterogeneous, context-dependent links that remain mechanistically plausible but not yet causally established. Since most individuals harbor several of these viruses simultaneously, their combined, possibly additive or synergistic, persistence over the lifespan may be more relevant to inflammaging than the effect of any single virus, making herpesvirus burden as a composite, rather than individual infections in isolation, a potentially more informative framework for understanding their role in aging.

Beyond the question of whether individual herpesviruses actively drive inflammaging, an equally compelling interpretation is that herpesvirus control itself may function as a dynamic readout of immune competence during aging. The progressive decline in VZV-specific CD4^+^ immunity that precedes zoster, the memory inflation that tracks CMV-specific T-cell exhaustion, and the counterintuitive decline in HSV reactivation frequency with age, despite presumed immunosenescence, together suggest that herpesvirus behavior reflects, and may even index, the trajectory of an individual’s immune aging more reliably than it reveals a single causal pathway.

Long-term antiviral therapy is already used to suppress recurrent HSV infection, and observational evidence suggests that anti-herpetic treatment may be associated with a reduced risk of dementia [92,121,122], although whether it modifies biological aging or extends lifespan remains unknown. Similarly, the established efficacy of zoster vaccination in preventing herpes zoster provides an opportunity to test whether preventing reactivation yields benefits beyond averting acute disease. These observations should be viewed as opportunities for testing causality, not as evidence that herpesvirus-targeted interventions are established anti-aging strategies.

Future research should prioritize prospective longitudinal cohorts pairing herpesvirus serostatus and biomarkers of viral activity with validated inflammaging measures to establish temporal relationships and distinguish active viral contributions from changes that simply accompany immune aging; causal inference approaches to determine whether persistent infection drives inflammaging or merely reflects declining immune control; mechanistic studies integrating virological, immunological, metabolic, and tissue-level measurements; and interventional studies with existing antivirals and vaccines, including zoster and future CMV vaccines, to test whether reducing viral persistence or reactivation measurably alters inflammaging and downstream age-related outcomes.

Taken together, the evidence supports a cautious view of herpesviruses as potential modifiers of aging trajectories rather than primary drivers, with a contribution likely to depend on cumulative exposure, host immune status, tissue context, and biological stage of aging. Determining whether, when, and in whom persistent herpesvirus infection becomes causally meaningful, and whether this contribution can be therapeutically modified, remains an important challenge for future research, one in which herpesviruses may prove as valuable for reading the aging immune system as for treating it.

## Figures and Tables

**Figure 1:**
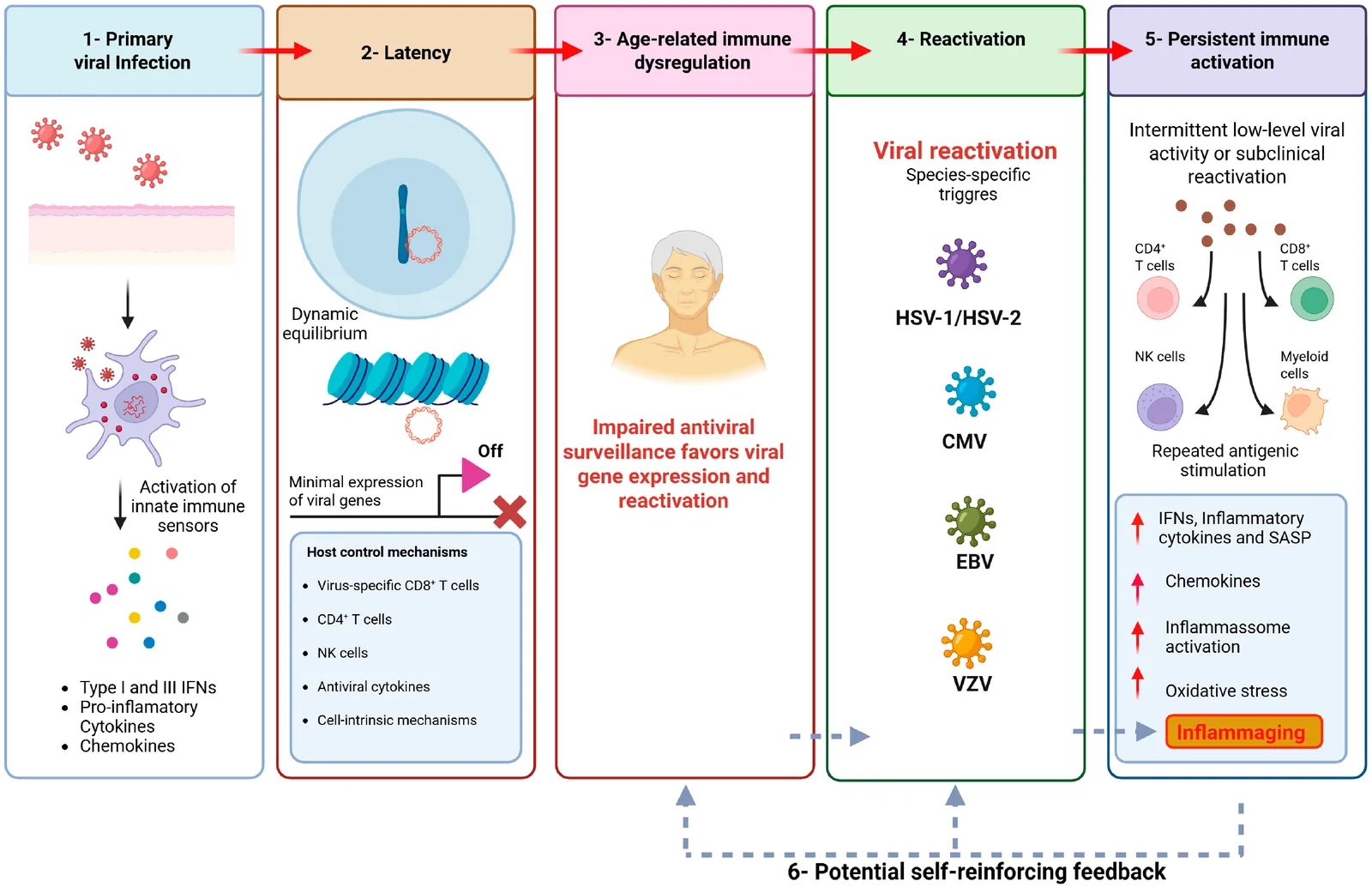
Conceptual model of herpesvirus persistence, immune aging, and inflammaging. Red arrows indicate the linear progression of herpesvirus infection, latency, and reactivation. (1) Primary herpesvirus infection establishes lifelong persistent infection, with viral genomes maintained in cell and tissue-specific reservoirs during latency and limited viral gene expression. (2–4) With advanced age, progressive changes in antiviral immune surveillance may reduce effective control of latent herpesviruses (HSV1, HSV2, CMV, EBV and VZV) and increase susceptibility to episodic or subclinical viral reactivation. (5) This may induce or amplify interferons, inflammatory cytokines and chemokines, SASP, and oxidative stress, thereby contributing to a chronic low-grade inflammatory milieu characteristic of inflammaging. (6) A potential self-reinforcing host–virus loop (dashed arrows) represents a proposed mechanistic context-dependent framework that age associated immune dysfunction may further compromise viral control. The model proposes herpesviruses as potential modulators of inflammaging rather than established causes of inflammaging.

**Figure 2:**
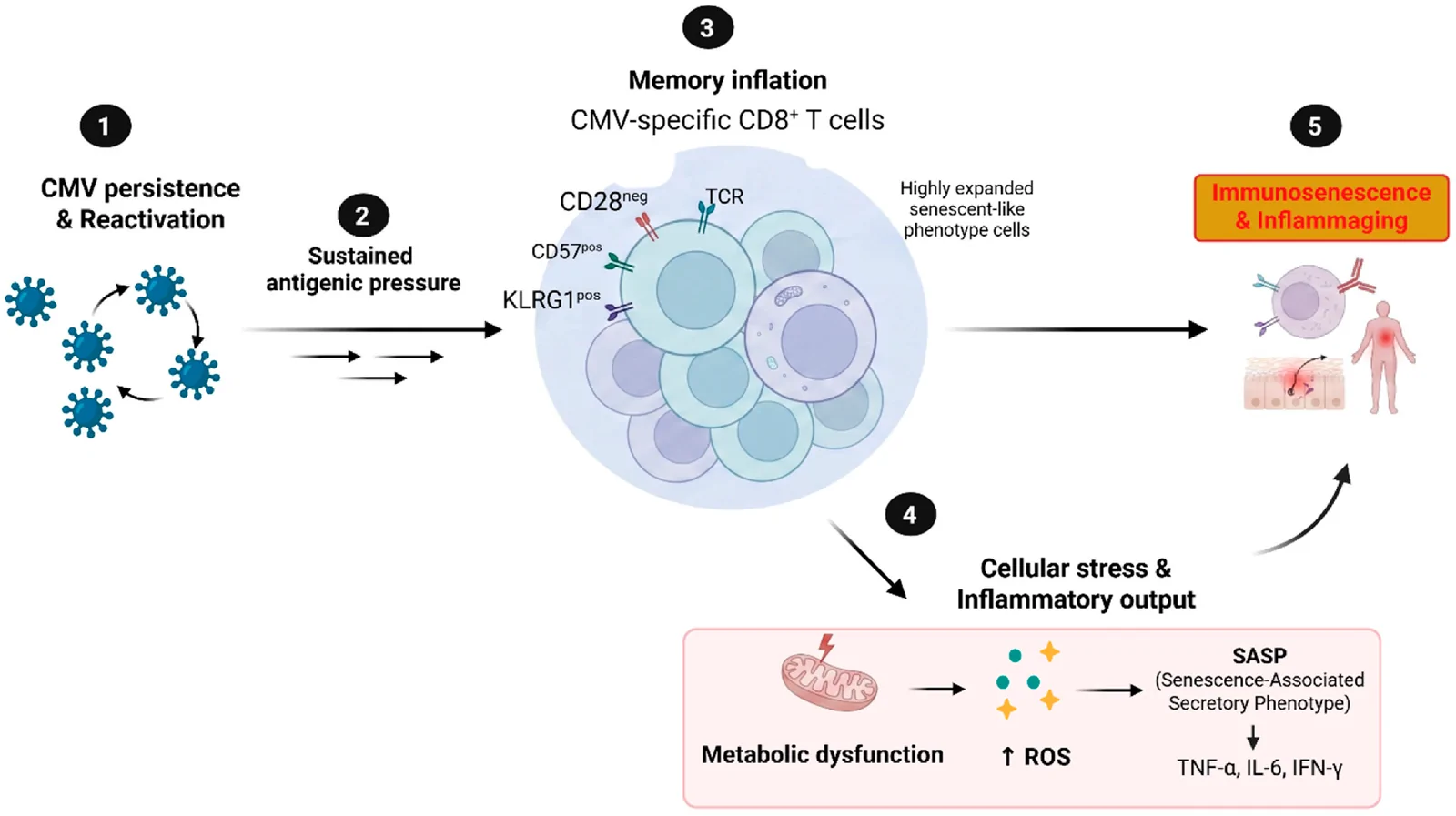
CMV persistence, antigenic stimulation and intermittent reactivation. (1) Persistent CMV infection and recurrent reactivation generate (2) sustained antigenic pressure, promoting (3) the accumulation and phenotypic remodeling of CMV-specific CD8^+^ T-cells, a process known as memory inflation. This phenotype is characterized by increased expression of differentiation and senescence-associated markers, including CD57 and KLRG1, together with loss of CD28. (4) Persistent immune activation may be accompanied by metabolic remodeling and cellular stress, including mitochondrial dysfunction, increased reactive oxygen species (ROS), and a shift toward glycolytic metabolism, potentially promoting a senescence-associated secretory phenotype (SASP) and the production of pro-inflammatory mediators such as TNF-α, IL-6, and IFN-γ. Collectively, these interconnected processes may contribute to (5) immunosenescence and chronic low-grade inflammation (inflammaging). The figure represents a proposed mechanistic model, and the causal relationships among these processes remain incompletely established.

**Figure 3:**
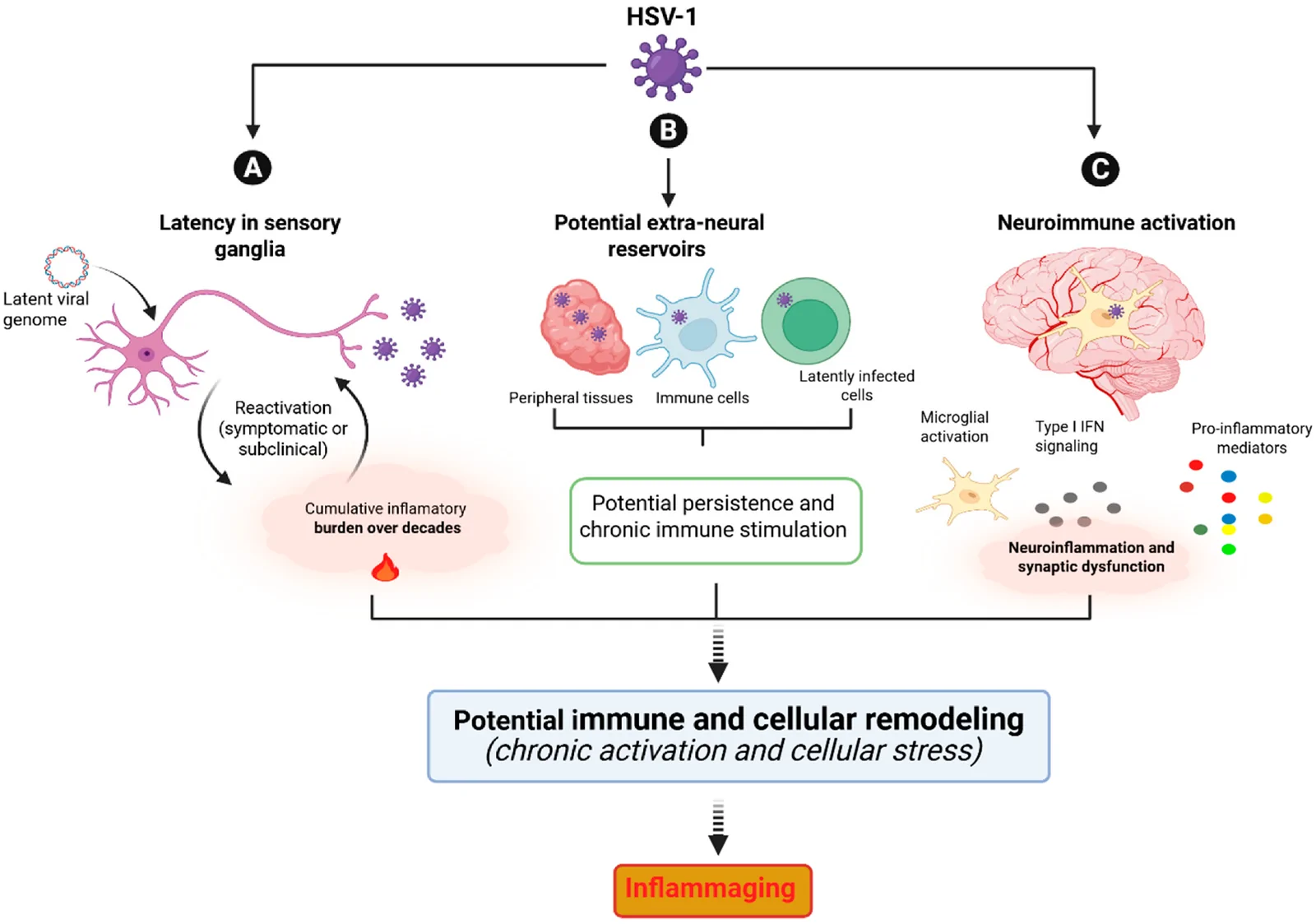
Potential mechanisms linking HSV-1 persistence to inflammaging. (**A**) HSV-1 establishes latency in sensory ganglia, with intermittent symptomatic or subclinical reactivation that may contribute to a cumulative inflammatory burden over time. (**B**) Potential extra-neural reservoirs may provide additional sites of viral persistence, although their contribution to long-term inflammation remains to be established. (**C**) HSV-1 may promote neuroimmune activation, including microglial activation, type I interferon signaling, and production of pro-inflammatory mediators, potentially contributing to neuroinflammation and synaptic dysfunction. These processes may converge on mitochondrial dysfunction and metabolic stress, innate immune activation, cellular stress and senescence, chronic production of pro-inflammatory mediators, and neuronal injury or loss of homeostasis, collectively contributing to chronic low-grade inflammation (inflammaging). Dashed lines indicate mechanisms that have not yet been fully characterized.

**Figure 4:**
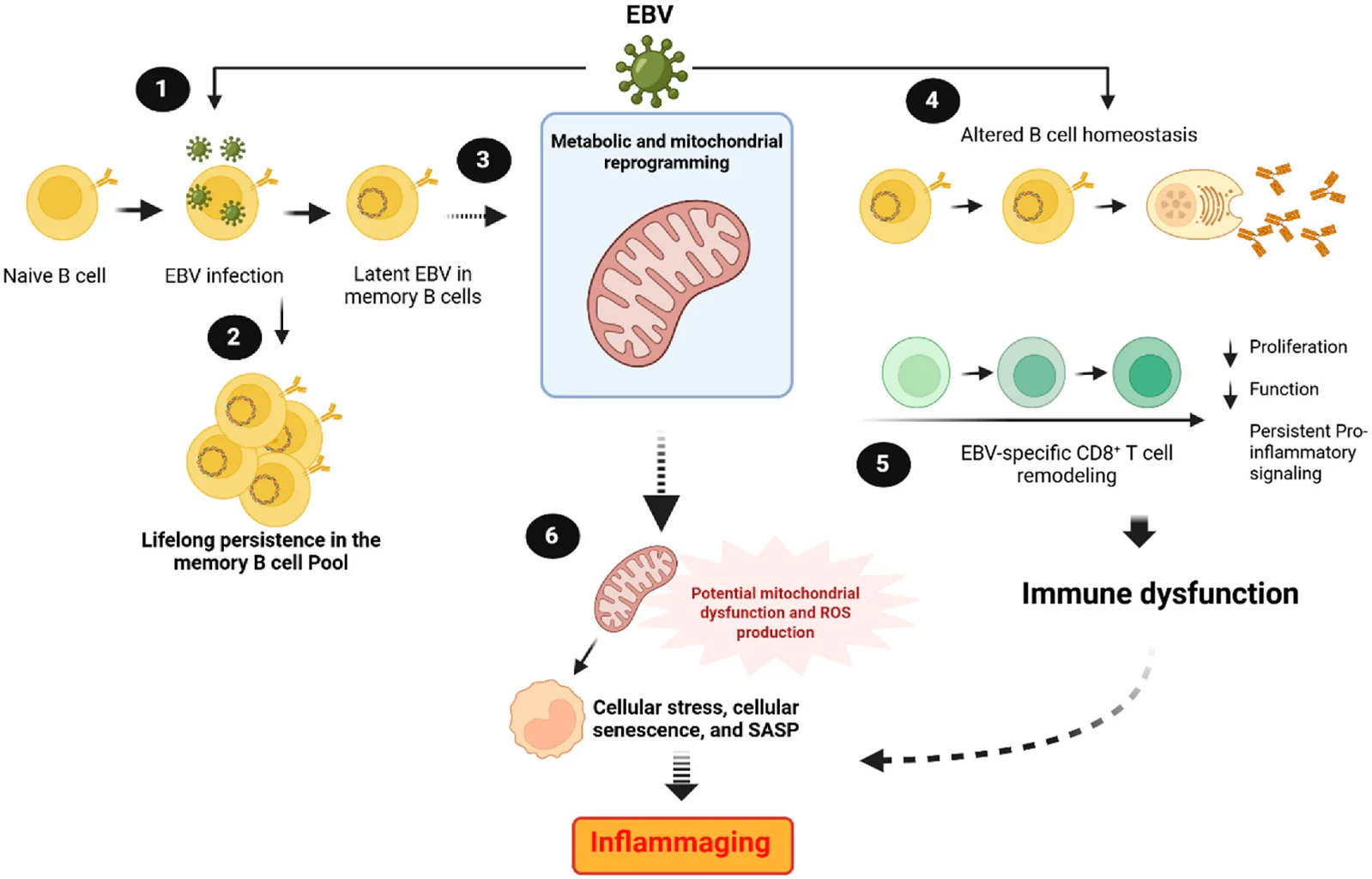
Potential mechanisms linking EBV persistence to inflammaging. (1) EBV infection of B-cells establishes latency, leading to (2) lifelong persistence within the memory B-cell. (3) Latent EBV infection may induce metabolic reprogramming and mitochondrial remodeling. (4) Persistent EBV may alter B-cell homeostasis, while (5) chronic antigenic stimulation and immune remodeling may affect CD8^+^ T-cell proliferation, differentiation, and function. Collectively, these processes may contribute to (6) immune dysfunction, cellular stress, senescence, and chronic low-grade inflammation associated with aging (inflammaging). The figure represents a proposed mechanistic framework and the extent to which persistent EBV independently contributes to systemic inflammaging and causal relationships between the depicted pathways remains incompletely established. Solid arrows represent evidence-supported relationships and dashed arrows represent proposed or potential bidirectional relationships.

**Figure 5:**
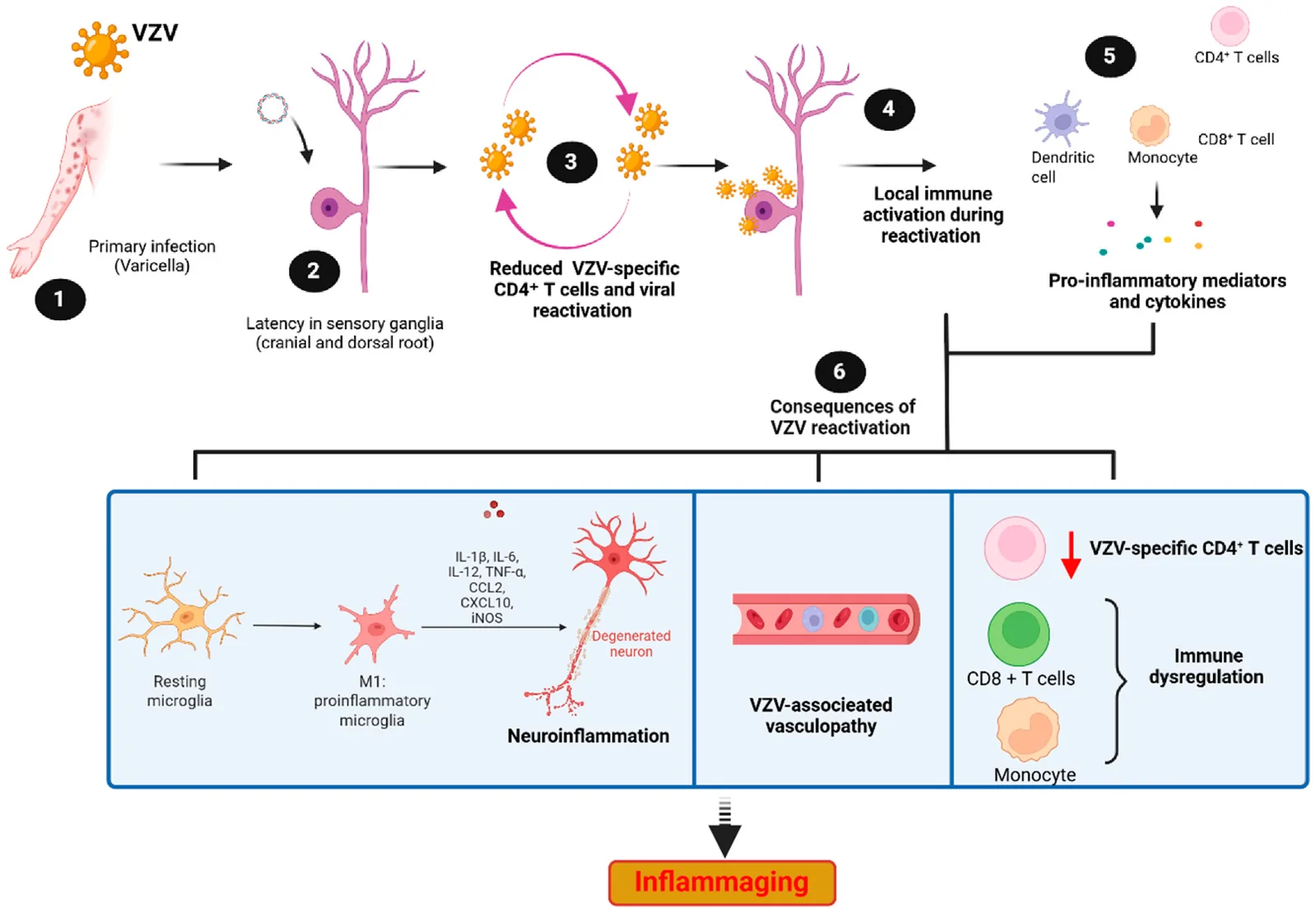
Potential mechanisms linking VZV reactivation to inflammaging. (1) Primary VZV infection (varicella) is followed by (2) lifelong latency in sensory ganglia. (3) Age-related decline in VZV-specific CD4^+^ T-cell frequency, function, and proliferation may increase the risk of intermittent viral reactivation. (4) Reactivation induces local immune activation and (5) recruitment of innate and adaptive immune cells, leading to the production of inflammatory mediators. (6) Recurrent VZV reactivation may contribute to: tissue inflammation, neuroinflammation, microglial activation, neuronal dysfunction, and VZV-associated vasculopathy, potentially increasing the long-term risk of cognitive decline. Collectively, these processes may contribute to chronic low-grade inflammation (inflammaging). Dashed lines indicate mechanisms that have not yet been fully characterized.

## Data Availability

No new data were created or analyzed in this study. Data sharing is not applicable to this article
